# Part 1—Cardiac Rehabilitation After an Acute Myocardial Infarction: Four Phases of the Programme—Where Do We Stand?

**DOI:** 10.3390/jcm14041117

**Published:** 2025-02-09

**Authors:** Aneta Aleksova, Alessandra Lucia Fluca, Antonio Paolo Beltrami, Elena Dozio, Gianfranco Sinagra, Maria Marketou, Milijana Janjusevic

**Affiliations:** 1 Cardiothoracovascular Department, Azienda Sanitaria Universitaria Giuliano Isontina, 34100 Trieste, Italy; alessandralucia.fluca@units.it (A.L.F.); gianfranco.sinagra@asugi.sanita.fvg.it (G.S.); mjanjusevic@units.it (M.J.); 2 Department of Medical Surgical and Health Sciences, University of Trieste, 34125 Trieste, Italy; 3 Dipartimento di Area Medica (DAME), Istituto di Patologia Clinica, University of Udine, 33100 Udine, Italy; antonio.beltrami@uniud.it; 4 Department of Biomedical Sciences for Health, University of Milan, 20122 Milan, Italy; elena.dozio@unimi.it; 5 Cardiology Department Crete, School of Medicine, Heraklion University General Hospital, University of Crete, 700 13 Heraklion, Greece; maryemarke@yahoo.gr

**Keywords:** cardiac rehabilitation, myocardial infarction, cardiac remodeling, mortality, illness perception

## Abstract

Cardiac rehabilitation is a well-established multidisciplinary interventional protocol that plays a pivotal role in the management and prevention of future cardiovascular events in patients with cardiovascular diseases. This patient-tailored approach includes educating patients about their cardiovascular condition and how to control the associated risk factors, an expert-designed lifestyle modification plan that may include exercise, proper nutrition, pharmacological treatment, and psychological support at each step. Exercise training represents a fundamental component of cardiac rehabilitation. It facilitates an enhancement of cardiovascular fitness, a reduction in heart rate, blood pressure and cardiac remodeling, an increase in the left ventricular ejection fraction, the optimization of endothelial function, and a reduction in inflammation and oxidative stress. Moreover, the beneficial physiological changes resulting from cardiac rehabilitation contribute to a reduction in morbidity and mortality in survivors of myocardial infarction (MI). Furthermore, the European Society of Cardiology Guidelines advocate for the initiation of cardiac rehabilitation as early as possible, while the patient who survived MI is still in hospital. This two-part comprehensive review commences with a historical overview of cardiac rehabilitation, followed by a detailed exploration of the four phases of the cardiac rehabilitation programme and its impact on cardiovascular health. In Part 2, the study aims to provide a detailed account of the optimal timing for starting cardiac rehabilitation programs and to examine the factors affecting low engagement in such programs, as well as gender-based differences in adherence.

## 1. Introduction

Acute coronary syndrome (ACS) represents an umbrella term for conditions caused by insufficient blood supply to the heart muscle, typically resulting from coronary artery disease (CAD) [1]. It encompasses unstable angina, non-ST-segment elevation myocardial infarction (NSTEMI), and ST-segment elevation myocardial infarction (STEMI) [1].

In recent decades, prompt myocardial reperfusion procedures and adequate medical therapy, as well as advances in medicine, have drastically improved the outcomes of patients with acute myocardial infarction (MI). However, this disease remains a significant global health problem. MI has a high prevalence and carries a high risk of morbidity and mortality [2]. Indeed, epidemiological studies have noted that ischemic heart disease affected approximately 126 million people worldwide in 2017. In addition, by observing its regional distribution, the highest prevalence of this disease is in Central and Eastern Europe, while Western Europe has shown an increasing prevalence over the last two decades [3].

After an acute MI, patients are susceptible to future complications such as recurrent MI, arrhythmias, stroke, and an overall decline in cardiac function, which may lead to heart failure (HF) [1]. The associated mortality risk is significant and varies depending on various factors such as the type of ACS, the extent of coronary artery involvement, the timeliness and effectiveness of medical intervention, and the presence of comorbidities such as hypertension, dyslipidemia, diabetes mellitus, a high body mass index and/or smoking habits [1]. It is estimated that acute MI accounts for 13.6% of in-hospital deaths and 10% of deaths within the first year of hospital discharge [1]. Moreover, according to the World Health Organization, in 2019, ACS was identified as the leading cause of mortality, being responsible for 16% of the world’s total deaths. Furthermore, during the period from 2000 to 2019, it was estimated that the greatest increase in mortality was attributable to ACS, with the number of cases rising from over 2 million to 8.9 million [4].

As recommended by the European Society of Cardiology Guidelines and the recent statement by the American Heart Association and the American Association of Cardiovascular and Pulmonary Rehabilitation, lifestyle changes and cardiac rehabilitation are effective approaches to mitigating cardiovascular risk factors and reducing the incidence of future events and poor outcomes [1,5]. However, there are still some critical aspects that need to be addressed. In particular, the low rates of referral to and participation in rehabilitation programs still represent a challenge [6]. Moreover, although cardiac rehabilitation promotes a healthier lifestyle, it is not uncommon for patients to return to previous habits after completing the process [1]. This is because patients often underestimate the real importance of certain cardiovascular risk factors. For instance, despite the well-established links between a poor diet, smoking, physical inactivity and cardiovascular disease, many patients fail to recognize the profound impact these behaviors have on their health. This lack of awareness or understanding often results in resistance to adopting healthier dietary patterns, engaging in regular physical activity or quitting smoking, even when explicitly advised to do so by healthcare providers. This discrepancy between perceived and actual risk has the effect of undermining preventive efforts and contributing to the persistence of modifiable risk factors, which in turn increases the burden of cardiovascular disease. This underscores the urgent need for the development of more effective strategies for the management of cardiovascular health post-MI [1]. Therefore, the physician cannot be underestimated, as they can improve adherence to rehabilitation programs and sustain engagement after their completion [1]. In addition, innovative strategies are needed to maintain patient interest in this secondary prevention measure [6].

This two-part review aims to provide a comprehensive overview of cardiac rehabilitation. More precisely, Part 1 explores the evolution and the current science of cardiac rehabilitation, explaining in detail the four phases of the programme, its clinical application and its benefits. In Part 2, we examined the optimal timing for the commencement of cardiac rehabilitation and the factors contributing to low participation in such programs, with a particular emphasis on the gender-based disparities in adherence.

## 2. A Historical Overview of Cardiac Rehabilitation in Post-MI Settings

The concept of physical activity for patients after an acute MI has transformed drastically over the past century. Initially, it was believed that bed rest and strict recumbency should be the standard treatment for patients who had recently survived a heart attack, as it was thought to reduce the risk of further complications such as arrhythmia, thrombosis or coronary ischemia [7,8,9]. It was assumed that the complex pathways associated with any form of physical activity would result in a redirection of energy, which was vital for the optimal healing of the heart [8]. To elucidate this point further, it is instructive to consider a noteworthy study from 1929 where bedrest for four to six weeks was recommended as a part of the treatment for thrombosis [7]. Over time, a slight transformation in the concept of lying in bed can be observed, as evidenced by a study conducted in 1952. The authors proposed a therapeutic approach that involves keeping the patient seated in a chair for extended periods, rather than resting him in bed [8]. This approach was designated as “armchair” therapy. The rationale for this treatment was based on emerging evidence at the time that strict bed rest could be harmful to patients with congestive HF. Consequently, the “armchair” position was believed to reduce the risk of lung congestion and pulmonary oedema.

As scientific knowledge expanded and numerous clinical research studies were conducted in the last century, it became increasingly evident that prolonged immobilization is highly dangerous, and that cardiac rehabilitation is an effective approach to mitigating the risk of morbidity and mortality. More precisely, prolonged bed rest is associated with a heightened risk of adverse in-hospital outcomes, including an elevated inflammatory state, the development of pressure ulcers and skin breakdown, calcium excretion, contracture and nerve injury. In conjunction with acute illness, bed rest and immobilization result in a rapid loss of bone and skeletal muscle mass within hours of hospital admission [10]. On the other hand, the early mobilization of patients during their hospitalization has been demonstrated to alleviate symptoms, thereby reducing the effects of bed rest in terms of regaining muscle strength, preventing contractures and maintaining joint flexibility. Furthermore, it has been shown to reduce the length of hospital stay, reduce hospital costs and improve patient outcomes [9,11,12,13].

It is interesting to note that the first randomized clinical trials of cardiac rehabilitation programs including patients after an MI, conducted between 1972 and 1985, demonstrated a reduction in mortality in treated patients, including studies with a follow-up of two to five years. However, this reduction was not statistically significant. Only one trial demonstrated a statistically significant reduction in mortality in the rehabilitation group. Therefore, in order to overcome the problem of not being able to statistically prove the benefits of cardiac rehabilitation programs, a meta-analysis that included ten randomized clinical trials involving 4347 patients was conducted in 1988. Of these, 2202 were randomized to a cardiac rehabilitation programme, while 2145 were assigned to the control group. The analysis demonstrated that the mortality risk was significantly lower in the rehabilitation group than in the control group, with a reduction in mortality of 25% [14]. Over the past few decades, numerous studies have been conducted in order to demonstrate the positive effects of cardiac rehabilitation and its early implementation among individuals who have survived an MI event, both STEMI and NSTEMI. These studies have concluded that cardiac rehabilitation strategies mitigate the mortality risk and risk of major cardiovascular events [15].

In addition, in light of the heightened susceptibility of the elderly to a deterioration in overall health subsequent to an MI event, it is recommended that cardiac rehabilitation be pursued as a particularly beneficial course of action for this cohort of patients. In a study that involved patients at least 70 years old who had experienced an MI or undergone one of a number of procedures, including coronary artery bypass surgery, heart valve surgery, or angioplasty, the aim was to ascertain changes over a three-week and six-week period following the event [16]. The study revealed that six weeks following the event, the overall disability of the participants increased in comparison to their pre-event status. Furthermore, the observed limitations in physical performance prior to discharge were found to be predictive of early disability in these patients. Depression was identified as one of the factors associated with a reduced physical performance and disability, together with other well-known comorbidities [16]. Therefore, helping older adults recover from a cardiac event requires effective strategies such as cardiac rehabilitation to reduce disability.

Finally, early mobility has been shown to improve patient outcomes, even in the intensive care unit. As soon as the cardiorespiratory test allows, early exercise should be introduced to reduce muscle dysfunction and other associated comorbidities. For example, in a study of patients with respiratory failure requiring mechanical ventilation, early mobilization was associated with a reduction in the length of hospital stay, followed by a reduced incidence of rehospitalization and mortality in the first year after discharge [17]. A further study examined the impact of bedside cycling on critically ill patients, commencing on day five. It was observed that the intervention was a safe and effective approach to preventing or mitigating declines in functional capacity [18]. More precisely, on the day of discharge from the hospital, patients who received treatment demonstrated superior performance on the six-minute walk test, exhibited higher quadriceps isometric strength and exhibited a more favorable subjective assessment of the functional state of their body [18]. Lastly, a meta-analysis from 2020 that was conducted by Zang et al. and included 15 randomized clinical trials and 1941 patients in total, examined the effects of early mobilization in intensive care units [19]. The findings indicated that early mobilization was an effective strategy for preventing the weakness that is commonly associated with a stay in the intensive care unit, reducing the length of hospital stay and improving functional mobility [19]. The recent CROS-II meta-analysis, which included randomized controlled trials and observational studies, reported a reduction in mortality in patients with acute coronary syndrome or after revascularization from 1995 onwards [20,21]. However, these results may be biased by the inclusion of observational studies. In contrast, in 2021, a meta-analysis that revised the Cochrane review included 85 randomized controlled trials involving 23,430 people with CHD [22]. At six to 12 months follow-up, cardiac rehabilitation was associated with a small reduction in all-cause mortality and a large reduction in MI and all-cause hospitalization [22]. At a follow-up of up to 12 months, the results were less clear, as cardiac rehabilitation was confirmed to have little or no effect on non-cardiovascular mortality and cardiovascular hospitalization [22]. These results are probably due to differences in the supply of cardiac rehabilitation, which can reasonably be attributed to the heterogeneity of clinical practice in the included studies, as they date from 1974 to 2020 [20].

## 3. Cardiac Rehabilitation

Therefore, as a result of numerous research studies conducted in the past, cardiac rehabilitation today is a well-established and safe, comprehensive, multidisciplinary and patient-tailored intervention that plays a critical role in the management and prevention of future cardiovascular events in all patients with cardiovascular disease in general [1,23].

The fundamental elements of cardiac rehabilitation encompass the initial assessment of the patient’s condition, the identification of the prevailing risk factors and the formulation of a strategy to mitigate them, followed by the education of patients regarding the advantages of physical activity, counselling and the creation of an exercise plan that the patient should adhere to, advice on nutrition, smoking cessation and psychosocial management, and professional support [1,23]. Finally, the objective of cardiac rehabilitation is to inform patients about the safe continuation of an individualized exercise programme and dietary recommendations following the completion of the programme in order to reduce the incidence of future cardiovascular events [23].

In practice, cardiac rehabilitation is divided into four main phases depending on the timing of their initiation. Phase I commences in the acute stage, while patients are still in hospital, and it is followed by Phase II, which takes place early after discharge. Phase III begins for outpatients and consists of creating a tailored exercise programme coupled with the control of cardiovascular risk factors. Finally, the aim of Phase IV is the maintenance of the improvements achieved [24,25]. A brief summary of the key findings observed in the studies discussed in this review is presented in Table 1.

### 3.1. Phase I Cardiac Rehabilitation

Phase I is the initial phase of cardiac rehabilitation, which is usually carried out while the patient is still in hospital [1,23]. In order to create a personalized cardiac rehabilitation plan and programme, health professionals assess the patient’s condition on the basis of the clinical–instrumental parameters obtained on admission and during the hospital stay, the medical procedures carried out, the treatments received on admission and during hospitalization, the patient’s previous clinical history and, finally, their family history [23]. In addition to these factors, the medical professionals will also consider the parameters they have obtained from the patient’s upper and lower extremity function tests, as well as the patient’s functional mobility, such as their ability to walk and perform self-care tasks [23].

In this initial phase, it is of the utmost importance to educate the patients about their condition and the benefits of exercise training for cardiovascular health, despite misconceptions to the contrary, and provide guidance on how to control modifiable and non-modifiable cardiovascular risk factors in order to ensure high-rate adherence to the programs [23,31]. A lack of understanding of the concept of cardiac rehabilitation was identified as one of the barriers to patient recruitment and adherence [31].

Regarding the physical aspects of cardiac rehabilitation, physical therapists should be engaged to design an exercise programme that is initially limited in terms of duration and intensity but gradually becomes more difficult and challenging in order to facilitate the patient’s return to full mobility. This process may initially entail merely sitting up in bed and/or monitoring patients’ ability to perform basic tests such as upper extremity and lower extremity function tests. Subsequent steps may then include progressing to short walks around the hospital. All the aforementioned in-hospital actions are meticulously tailored to each patient separately and are monitored carefully [32]. Finally, patients should be informed of how to manage the psychological aspects of the event, pain or other symptoms [23,32].

Concerning the precise point at which patients should commence Phase I of the cardiac rehabilitation programme, a number of studies have demonstrated that this should be done as soon as possible. A study conducted by Zheng et al. in 2008 demonstrated that initiating supervised exercise training in patients admitted for acute MI within only three to seven days post-PCI could ameliorate the patient’s condition. More precisely, in this single-blind, randomized controlled trial, the researchers randomly assigned 60 patients to two groups: one that engaged in a six-month exercise program three times a week with supervision from a cardiologist and a nurse, and another that received pharmacological therapy and standard advice regarding lifestyle modifications in order to ameliorate their condition [11]. The study showed that exercise training as a part of cardiac rehabilitation was associated with a reduction in cardiac remodeling and an increase in the left ventricular ejection fraction (LVEF) [11]. Another study also confirmed that a six-month supervised exercise programme administered within a week of PCI in patients admitted for acute MI resulted in markedly elevated levels of LVEF and a discernibly enhanced quality of life in comparison to the control group [12]. In 2021, Nakamura et al. demonstrated that early rehabilitation performed within three days was associated with a shorter hospital stay, thus corroborating the function [13]. Interestingly, no evident changes in the grade of autonomy were observed when comparing the group receiving early rehabilitation and the one with usual care [13].

However, the effects of inpatient rehabilitation on prognosis are a topic of ongoing investigation. According to the findings of Kanazawa et al. from 2020, Phase I rehabilitation within several days of admission was effective in reducing the risk of revascularization by 20% and readmissions for cardiac disease by 19% over an average follow-up of about one year [26]. Based on the authors’ findings, there was a dose-dependent association with a reduction in risk for the investigated outcomes; however, low-frequency rehabilitation also leads to better outcomes [26].

**Figure 1:**
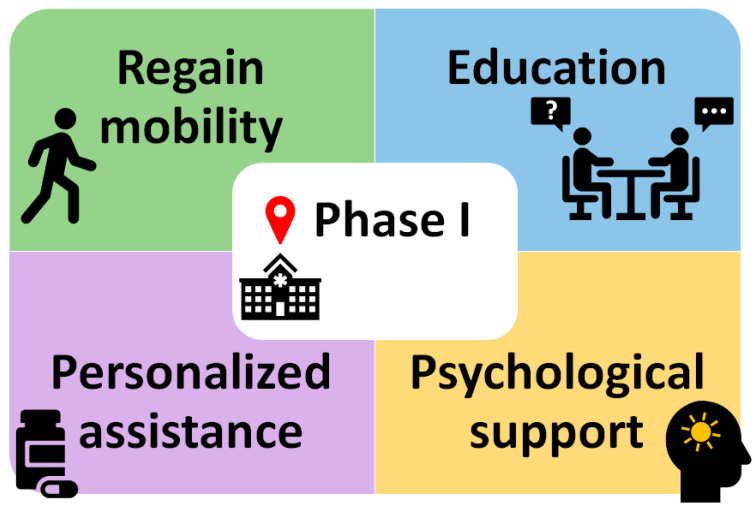
Components of Phase I of cardiac rehabilitation.

Finally, in addition to advice and a tailor-made programme to reduce risk factors through lifestyle changes such as an exercise plan, appropriate diet and pharmacological treatment, it is also very important to provide patients with psychosocial support. Acute MI can have a significant psychological impact on patients, leading to disorders such as anxiety and depression that ultimately reduce their quality of life. Therefore, it is important to address this aspect and provide psychosocial support to patients, offering professional counselling and stress management techniques (Figure 1) [33].

### 3.2. Phase II Cardiac Rehabilitation

The phase following discharge is referred to as Phase II [34]. In Europe, the duration of Phase II typically ranges from 6 to 12 weeks, with some programs extending from 2 to 24 weeks, depending on the modality of delivery, namely whether the programme is provided as an inpatient, outpatient, or home-based programme. In the United States, the duration of an outpatient programme is contingent upon the insurance coverage and the health insurance provider. The duration of such programs varies, with a range of 6 to 36 visits over a period of 2 to 18 weeks [30]. The objective of this phase is to implement all the recommendations of healthcare professionals during the patient’s period of hospitalization [32]. At this stage, the patient must remain under the supervision of a qualified professional to ensure the proper implementation of lifestyle changes and the progression of their exercise regimen. In addition, patients should learn how to monitor their heart rate and effort levels during exercise, as this will facilitate their independence and allow them to progress to Phase III [32].

**Figure 2:**
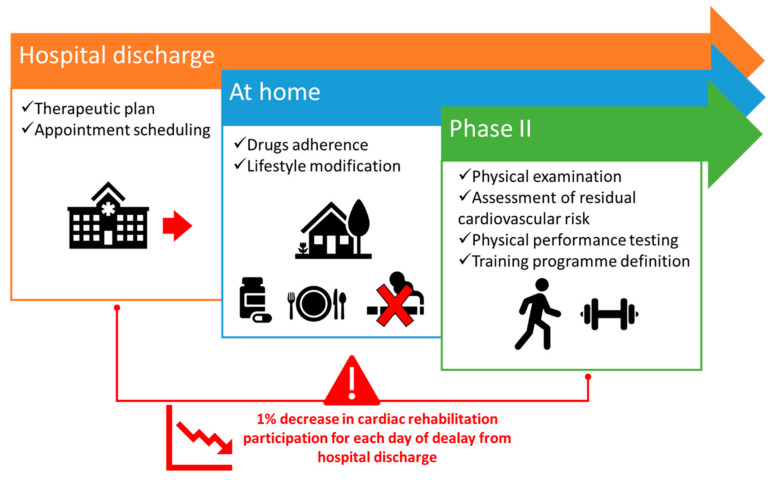
Events following hospital discharge.

In the event that cardiac rehabilitation has not commenced within the hospital setting within a few days of the acute ischemic event (with Phase I), and in the event that patients have not been discharged with a pre-established cardiac rehabilitation plan, which is the most common case in clinical practice, Phase II commences with the scheduling of an appointment for the evaluation of patients’ psychophysical status after myocardial infarction. It has been estimated that the optimal time for the initial consultation is between one and four weeks. This high variability is primarily attributable to the difficulty of scheduling appointments due to waiting times or concerns regarding the efficacy of the rehabilitative programme. In addition to a comprehensive physical examination, the appointment includes the assessment of residual cardiovascular risk, the evaluation of comorbidities and the adherence to medical treatment. Moreover, the physical performance of patients is tested using non-invasive strategies to investigate their exercise tolerance. Indeed, physical activity counselling provides insight into the patient’s capacity to sustain physical activity and guides the physician in selecting the most suitable training programme, which could be aerobic or strength exercise training (Figure 2). The former is defined as any activity that uses large muscle groups in a rhythmic manner, such as cycling, hiking and jogging [35]. The second implies the involvement of a single muscle group contraction against an external resistance, such as in weightlifting [36]. Both have been reported to have positive effects in the context of cardiac rehabilitation [37]. In particular, aerobic exercise improves cardiovascular risk factors such as hypertension, insulin resistance and obesity. Similarly, strength training has been demonstrated to ameliorate glucose metabolism and body composition. Furthermore, it increases the basal metabolic rate and muscle strength [37].

Besides considering the type of exercise, it is fundamental to evaluate some principles of exercise programme prescription, such as its frequency, intensity, time, volume and progression [38]. Frequency defines the number of times exercises are performed during the week, whereas intensity indicates the energy required to perform the activity, which is calibrated according to the initial assessment of patients’ characteristics considering calorie expenditure or oxygen consumption. The duration of the exercise session is expressed by the time variable [38]. Meanwhile, the volume is the product of frequency, intensity and time, and provides the total amount of exercise [38]. Finally, when defining the exercise programme, it is important to consider periodic adjustments to the training in order to ensure that it is adapted to the patient’s progression towards physical activity [37].

**Figure 3:**
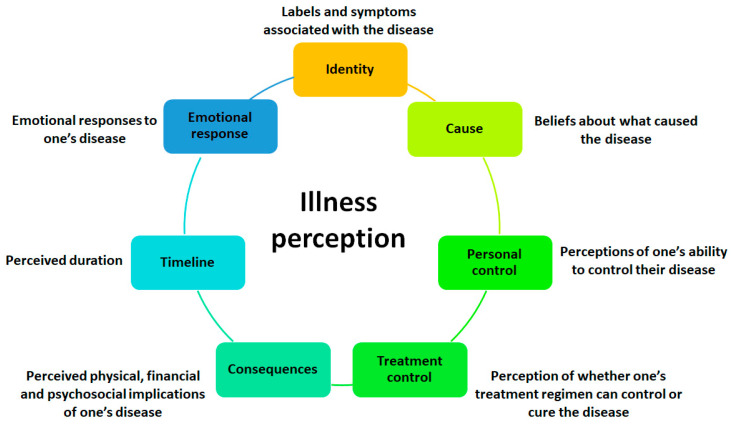
Main features of illness perception.

It is important to emphasize that the implementation of an optimal rehabilitation programme is not in itself sufficient to achieve the goals of secondary prevention. According to data in the literature, patients who have experienced an acute MI and understand their clinical condition express a higher motivation to maintain long-term control over the pathology [39]. This concept is summarized by the term “illness perception” and is a fundamental aspect that allows adaptation to changes in daily life following acute MI. The perception of illness is characterized by several factors, including the way in which the person describes their clinical condition, the perception of the causes of the disease and its duration, the degree of control the patient has over the treatment and the course of the disease, and the emotional consequences (Figure 3) [28].

Here, in this context, it is worth mentioning an interesting study published in 2023 by Darsin Singh et al. that evaluated the impact of cardiac rehabilitation on the perception of the disease [28]. A total of 450 patients completed the Brief Illness Perception Questionnaire (BIPQ) to assess the components of illness perception at three time points: before the start of rehabilitation, after four sessions, and after eight sessions. The authors observed an overall improvement in illness perception between baseline and the first time point, followed by a reduction in indicators at the second time point. The findings indicate a greater understanding of ACS, which, when combined with an awareness of the chronicity of the disease and the experience of everyday symptoms, raises concerns that significantly influence the emotional state of patients. Therefore, the decline observed during the second time point can be explained by the fact that patients may perceive their health status as unsatisfactory despite rehabilitation [28]. Consequently, the perception of illness is susceptible to change, and if such changes occur during Phase II of rehabilitation, they can eventually compromise adherence. Furthermore, patients who exhibited more severe symptoms or had undergone surgery were more likely to engage in rehabilitation, likely due to a more comprehensive understanding of the multifaceted nature of the illness [28].

It is also noteworthy that commencing the cardiac rehabilitation protocol with Phase II following discharge results in favorable outcomes in terms of enhanced patient well-being. For instance, in a study that randomly divided patients with acute MI into two groups, the first group of patients was prescribed conventional medical therapy, while the second group was assigned to a three-month exercise programme after discharge, followed by a community-based self-managed programme for a period of nine months [27]. The study demonstrated that patients in the rehabilitation group had a significantly lower risk of major adverse cardiovascular events (MACEs), better left ventricular systolic function, and better results on the six-minute walking distance test compared to the control group. This indicates that there has been an improvement in overall health, cardiac function and physical stamina in general due to the implementation of the protocol [27].

### 3.3. Phase III and Phase IV Cardiac Rehabilitation

Patients who complete Phase II of cardiac rehabilitation are strongly encouraged to continue with Phase III. The objective of Phase III is to facilitate long-term adherence to a tailored exercise programme and the other positive lifestyle changes implemented during Phase II, with the aim of mitigating cardiovascular risk factors [30]. Phase III cardiac rehabilitation programs are overseen by healthcare professionals, although the level of supervision is less rigorous than that provided in Phase II [30]. Finally, Phase IV, a “maintenance phase” of cardiac rehabilitation, is the final stage [24]. It is intended for patients who have successfully completed the earlier phases and are now focusing on maintaining the progress achieved and preventing future cardiac events through a long-term commitment to a healthy heart lifestyle [24].

Unfortunately, there is a paucity of literature regarding the beneficial effects of Phase III and Phase IV. Only a few studies have addressed this question, and the results are modest. A retrospective study from 2017 by Brawner et al. comprising 230 patients who commenced the Phase III cardiac rehabilitation programme for eight weeks, both regularly and irregularly, demonstrated that Phase III had no beneficial effect on clinical events such as non-fatal MI or HF hospitalization during the long-term follow-up period of 5.6 years [30]. On the other hand, another study comprising 3241 patients who had survived an MI indicated that adherence to a three-year multifactorial educational and behavioral programme was associated with a reduced incidence of cardiac events in comparison to the control group [29]. From these data, it might be concluded that patients usually revert to their previous unhealthy habits following the completion of the programme, which could impair the beneficial long-lasting effects of cardiac rehabilitation. Conversely, continuous support might help maintain or enhance participants’ fitness levels and self-confidence, reduce anxiety and promote a positive outlook on health and the future. These psychological factors facilitate lifestyle modifications and improve overall cardiovascular health. Nevertheless, further studies are needed to explore the positive effects of Phase III and Phase IV in patients with MI.

### 3.4. Current and Emerging Trials of Cardiac Rehabilitation Including Patients with ACS

In recent years, several ongoing clinical trials have focused on evaluating cardiac rehabilitation programs specifically designed for patients who have experienced an AMI. These trials aim to improve our understanding of the effectiveness of different rehabilitation strategies and their impact on patient outcomes. In a double-blind randomized controlled trial, 24 individuals diagnosed with AMI will be randomly assigned to either an intervention or control group in a 1:1 ratio [2]. The intervention group will undergo a personalized, exercise-based cardiac rehabilitation program during hospitalization, followed by a semi-supervised regimen post-discharge. Meanwhile, the control group will receive standard medical care. Key outcome measures will include cardiac remodeling (evaluated via cardiac magnetic resonance imaging), functional capacity (measured through maximal oxygen consumption), and cardiac autonomic function (analyzed using heart rate variability). Secondary assessments will focus on safety and the total volume of exercise completed throughout the study [2]. It is also important to mention another study here, namely the MCNAIR Study (coMparative effeCtiveness of iN-person and teleheAlth cardIac Rehabilitation), which evaluates two approaches to the delivery of cardiac rehabilitation: traditional in-person sessions and remote delivery by means of telehealth [40]. Finally, it is worth mentioning the results from the recently published RESILIENT study [41]. The present randomized clinical trial is of significance as it examines the effects of mobile health-based cardiac rehabilitation (mHealth-CR) in older adults with ischemic heart disease, comparing it to standard care. The findings of the study indicated that mHealth-CR did not result in improvements in the patients’ conditions, as measured by changes in their 6 min walk distance, health status, or daily activities. These findings underscore the need for age-specific adaptations in mHealth strategies to enhance their effectiveness for older adults in order to effectively improve outcomes [41].

## 4. Conclusions

In conclusion, cardiac rehabilitation programs can reduce the risk of morbidity and mortality by optimizing cardiovascular health in patients after acute MI. This is achieved by educating patients on risk factor control and lifestyle modification, including exercise training, proper nutrition and pharmacological treatment, and by providing psychological support. Most importantly, the cardiac rehabilitation program is tailored to the individual condition of each patient. Although both the inpatient and outpatient settings of cardiac rehabilitation programs yield positive results in terms of reducing left ventricular remodeling, increasing left ventricular function, improving exercise capacity and endurance, and lowering rates of MACE, inpatient settings have been shown to also reduce patients’ bed rest time, hospital stay, and hospital costs. Nevertheless, despite the proven benefits, cardiac rehabilitation programs are underused for a variety of reasons, which are explained in detail in Part 2 of this two-part comprehensive review.

## Figures and Tables

**Table 1:** Key findings regarding the effects of cardiac rehabilitation in patients with myocardial infarction across different rehabilitation phases.

Author	Patients	Type of Study	Findings	Ref.
Phase I				
Zheng et al. (2008)	57 (27 Exercise group vs. 30 Control group)	RCT	↓ Cardiac remodeling ↑ LVEF	[11]
Kanazawa et al. (2020)	13,697 (4742 No rehabilitation vs. 8955 Rehabilitation)	Retrospective	↓ by 20% the risk of revascularization ↓ by 19% readmissions for cardiac disease Dose-dependent association with reduced risk for the investigated outcomes Low-frequency rehabilitation leads to better outcomes	[26]
Jiang et al. (2021)	98 (49 kinetic energy progressive exercise vs. 49 Routine intervention)	RCT	↑ LVEF ↑ Quality of life	[12]
Nakamura et al. (2021)	31,603 (26,456 Usual care vs. 5147 Early rehabilitation	Retrospective	↓ Hospital stay No changes in autonomy comparing the group with early rehabilitation and the one with usual care	[13]
Phase II				
Xiao et al. (2021)	164 (82 Rehabilitation programme + community-based exercise vs. 82 No rehabilitation)	RCT	↓ Risk of MACE ↑ LVEF ↑ Results on the six-minute walking distance test	[27]
Darsin Singh et al. (2023)	450 who underwent phase II rehabilitation programme	Descriptive longitudinal study	↑ Illness perception after cardiac rehabilitation Patients with worse symptoms or had undergone surgery were more likely to engage in rehabilitation	[28]
Phase III and IV				
Giannuzzi et al. (2008)	3241 (1620 Intervention group vs. 1621 Usual care group)	RCT	↓ Incidence of cardiac events	[29]
Brawner et al. (2017)	230 (101 Regular participants vs. 129 Irregular participants)	Retrospective	No beneficial effect on clinical events (non-fatal MI or HF hospitalization)	[30]

HF, heart failure; LVEF, left ventricular ejection fraction; MACE, major adverse cardiovascular events; MI, Myocardial infarction; RCT, Randomized Controlled Trial. explanations for the arrows: ↑: increase, ↓: decrease.
